# 直接抗人球蛋白试验阴性自身免疫性溶血性贫血患者临床特征及预后分析

**DOI:** 10.3760/cma.j.cn121090-20260507-00227

**Published:** 2026-08

**Authors:** 骏安 张, 洁雯 邱, 均 施, 凤奎 张, 以州 郑, 美丽 葛, 馨 赵

**Affiliations:** 1 中国医学科学院血液病医院（中国医学科学院血液学研究所），血液与健康全国重点实验室，血液系统疾病国家临床医学研究中心，细胞生态海河实验室，天津 300020 State Key Laboratory of Experimental Hematology, National Clinical Research Center for Blood Diseases, Institute of Hematology & Blood Diseases Hospital, Chinese Academy of Medical Sciences & Peking Union Medical College, Haihe Laboratory of Cell Ecosystem, Tianjin 300020, China; 2 天津医学健康研究院，天津 301600 Tianjin Institutes of Health Science, Tianjin 301600, China

**Keywords:** 贫血，溶血性，自身免疫性, 直接抗人球蛋白试验, 长期预后, 治疗模式, Anemia, hemolytic, autoimmune, Direct antiglobulin test, Long-term prognosis, Treatment patterns

## Abstract

**目的:**

探讨直接抗人球蛋白试验（Direct antiglobulin test，DAT）阴性自身免疫性溶血性贫血（Autoimmune hemolytic anemia，AIHA）的临床特征及转归，为该亚型的临床诊疗提供依据。

**方法:**

本研究为单中心回顾性队列研究。分析2014年1月至2023年12月中国医学科学院血液病医院收治的30例DAT阴性AIHA患者。入组病例经微柱凝胶法广谱及单特异性抗血清检测均呈阴性，并严格排除其他溶血性疾病。对患者基线实验室指标、治疗反应及转归进行分析。

**结果:**

共纳入30例DAT阴性AIHA患者，占同期AIHA住院病例的4.77％，其中女性20例（66.7％），男性10例（33.3％），原发性26例（86.7％），继发性4例（13.3％），确诊年龄（44.8±17.8）岁。起病后最低HGB为（61.3±15.9）g/L。一线糖皮质激素总缓解率达93.3％（28/30），二线治疗以利妥昔单抗为主。2例糖皮质激素无效者经伊布替尼+利妥昔单抗及环孢素+利妥昔单抗挽救获完全缓解。中位随访64个月，全因死亡率为10.0％（3/30），中位无事件生存（EFS）期17.0个月，1年、3年及5年EFS率分别为56.5％、45.9％和37.5％。复发诱因主要为药物减撤（73.7％）及感染（21.1％）。

**结论:**

在完善排除性诊断并排除IgA/IgM介导型后，DAT阴性AIHA预后相对良好，对糖皮质激素及利妥昔单抗等多种免疫抑制治疗反应相对理想。

## Abstract (en)

**Objective:**

To investigate the clinical characteristics and outcomes of direct antiglobulin test（DAT）-negative autoimmune hemolytic anemia（AIHA）and to provide evidence for the clinical diagnosis and management of this subtype.

**Methods:**

This was a single-center retrospective cohort study. Thirty patients with DAT-negative AIHA who were treated at the Institute of Hematology & Blood Diseases Hospital, Chinese Academy of Medical Sciences, between January 2014 and December 2023 were included. All included patients tested negative by the microcolumn gel method using both polyspecific and monospecific antiglobulin reagents, and those with other causes of hemolytic anemia were rigorously excluded. The patients' baseline laboratory parameters, outcomes, and treatment responses were analyzed.

**Results:**

A total of 30 patients with DAT-negative AIHA were included, accounting for 4.77％ of all patients hospitalized with AIHA during the same period; 20（66.7％）were female and 10（33.3％）were male; 26（86.7％）had primary AIHA and 4（13.3％）had secondary AIHA cases. The age at diagnosis was（44.8±17.8）years. The nadir hemoglobin level at disease onset was（61.3±15.9）g/L. The overall response rate to first-line glucocorticoid therapy was 93.3％（28/30）, and rituximab was the most commonly used second-line treatment. Both patients who failed to respond to glucocorticoids achieved remission following salvage treatment with ibrutinib plus rituximab and cyclosporine plus rituximab. At a median follow-up of 64 months, the all-cause mortality rate was 10.0％（3/30）. The median event-free survival（EFS）was 17.0 months, with 1-, 3-, and 5-year EFS rates of 56.5％, 45.9％, and 37.5％, respectively. Relapses were mainly triggered by drug tapering（73.7％）and infection（21.1％）.

**Conclusion:**

After a comprehensive diagnostic workup to exclude other causes and immunoglobulin A（IgA）- or IgM-mediated AIHA, patients with DAT-negative AIHA generally have a relatively favorable prognosis and show good responses to glucocorticoids, rituximab, and other immunosuppressive therapies.

自身免疫性溶血性贫血（autoimmune hemolytic anemia，AIHA）是一组异质性显著的获得性溶血性贫血，其特征为自身抗体介导的红细胞破坏速率超过了骨髓的代偿潜能。直接抗人球蛋白试验（direct antiglobulin test，DAT，又称Coombs试验）阳性长期被认为是诊断AIHA的基石之一。然而，临床上约5％的AIHA患者常规DAT检测结果呈阴性，这类病例被称为Coombs阴性或DAT阴性AIHA[1]–[2]。

常规DAT阴性可能与红细胞表面结合IgG数量低于检测阈值、抗体亲和力较低，以及存在IgA或IgM等常规试剂难以检出的抗体类型有关[3]。采用更敏感的DAT、流式细胞术以及针对低亲和力或特殊免疫球蛋白类型的检测方法，可识别部分常规DAT阴性的病例，但DAT阴性AIHA本质上仍属于排除性诊断，需结合临床资料审慎判断[4]。

DAT阴性AIHA较为少见，目前相关临床研究仍较有限。既往研究提示，DAT阴性AIHA在疾病严重程度、糖皮质激素治疗反应及预后等方面可能具有一定临床特点，但不同研究的检测方法和诊断标准并不完全一致，其临床表型及长期转归仍需进一步积累。为进一步总结DAT阴性AIHA的临床特征，本研究回顾性分析本中心30例DAT阴性AIHA患者的临床资料，分析其临床及实验室特征、治疗反应和随访转归，现报告如下。

## 病例与方法

一、病例资料

本研究采用单中心回顾性队列设计，纳入2014年1月至2023年12月于中国医学科学院血液病医院住院诊治的DAT阴性AIHA患者。入组标准：①存在明确的溶血性贫血临床及实验室证据，表现为不同程度的贫血、黄疸或脾大，HGB水平符合贫血诊断，结合珠蛋白（Hp）降低，血总胆红素（TBIL）升高且以非结合胆红素升高为主，乳酸脱氢酶升高伴网织红细胞比例（Ret％）或绝对值（ARC）升高；②DAT阴性：经微柱凝胶法筛查，并联合广谱（含IgA/IgM）多特异性及IgA/IgM单特异性抗血清复核，结果均呈阴性；③经系统性筛查排除其他溶血或黄疸性疾病：完善红细胞渗透脆性试验（EOFT）、红细胞酶谱（G6PD、PK、5′-NT）、阵发性睡眠性血红蛋白尿（PNH）克隆、血红蛋白电泳、造血原料、骨髓细胞形态学评估、核型分析等检测，并排除了骨髓增生异常综合征（MDS）、机械性溶血、先天性红细胞生成异常性贫血（CDA）、噬血细胞综合征、药物继发的AIHA及非溶血性黄疸等；④对至少一种免疫抑制治疗有明确应答：纳入的免疫抑制治疗包括糖皮质激素、利妥昔单抗［含标准剂量（RTX-SD，每周375 mg/m^2^×4周或1 000 mg/d，第1、15天）及低剂量（RTX-LD，每周100 mg×4周）］、环孢素、硫唑嘌呤、伊布替尼及氟达拉滨。

二、数据采集

收集患者基线人口统计学特征（性别、年龄、起病及确诊时间）及临床事件（复发频次与诱因、血栓类型及发生时间）。采集基线实验室指标包括：①全血细胞计数，含红细胞参数：HGB、红细胞比容（Hct）、平均红细胞体积（MCV）、平均红细胞血红蛋白量（MCH）及平均红细胞血红蛋白浓度（MCHC）；②溶血及生化指标：乳酸脱氢酶（LDH）、间接胆红素（IBIL）、Hp、血浆游离血红蛋白（F-Hb）、红细胞生成素（EPO）水平、酸化甘油溶血试验（AGLT50）及EOFT；③骨髓代偿指标：Ret％及ARC，并依据公式计算骨髓反应指数［BMRI＝ARC×（患者HGB / HGB正常下限）］及网织红细胞生成指数（RPI）[5]。此外，详细记录各线治疗方案、疗效及长期随访生存数据。本研究于2025年4月完成标准化统一随访。

三、标准及定义

贫血严重程度依据HGB水平划分为极重度（<30 g/L）、重度（30～<60 g/L）、中度（60～<90 g/L）及轻度（≥90 g/L且低于正常下限）。疗效判定：完全缓解（CR）定义为HGB≥120 g/L（男性）或≥110 g/L（女性）且不依赖输血；部分缓解（PR）定义为未达CR标准，但HGB较基线升高≥20 g/L或HGB≥100 g/L且不依赖输血；CR及PR统称为总缓解（OR）；无效（NR）为未达上述标准。预后指标：总生存（OS）期定义为确诊至死亡或随访截止日期的时间；无事件生存（EFS）期定义为确诊至首次复发、死亡或随访截止日期的时间；复发定义为排除失血等混杂因素后，HGB下降≥20 g/L。

四、统计学处理

数据分析及绘图采用SPSS 26.0及R 4.5.0软件。符合正态分布的计量资料以均值±标准差表示，非正态分布资料以中位数（范围）表示，计数资料以例数（百分比）表示。EFS及OS采用Kaplan-Meier法绘制生存曲线并计算生存率。

## 结果

一、一般特征

本研究共筛选629例AIHA患者，其中30例符合DAT阴性AIHA（4.77％），纳入本研究。其中女性20例（66.7％），男性10例（33.3％）。患者起病年龄为（43.1±17.8）岁，确诊年龄为（44.8±17.8）岁，从出现症状到确诊的平均病程为1.7年。原发性AIHA 26例（86.7％）；继发性AIHA 4例（13.3％），原发病包括自身免疫性疾病2例（类风湿性关节炎、抗磷脂综合征各1例）及淋巴增殖性疾病2例（轻链型意义未明单克隆免疫球蛋白血症、非霍奇金淋巴瘤各1例）。另有合并Gilbert综合征及未分化结缔组织病各1例。

二、实验室检查

全组患者获得首次OR前的最低HGB水平为（61.3±15.9）g/L，以中重度贫血为主，其中中度贫血16例（53.3％），重度贫血12例（40.0％），轻度贫血2例（6.7％）。治疗前基线平均HGB为（77.4±15.5）g/L，平均RBC为（2.18±0.50）×10^12^/L，平均Hct为（24.4±5.1）％。中位WBC为5.7（1.5～12.9）×10^9^/L，平均PLT为（185.5±69.8）×10^9^/L。红细胞参数呈大细胞性贫血特征，平均MCV为（113.2±12.6）fl，中位MCH为35.1（28.9～45.6）pg，平均MCHC为（317.4±16.2）g/L。溶血指标显著异常：中位LDH为322.2（240.0～540.1）U/L，中位TBIL为68.2（47.7～82.1）µmol/L，且以IBIL升高为主［中位IBIL 54.5（39.7～68.5）µmol/L］。F-Hb中位数为56.7（37.7～87.5）mg/L，Hp显著降低，中位数为0.185（0.125～0.375）g/L。EPO水平反应性升高，中位数为98.3（52.6～191.9）U/L。多数呈红细胞渗透脆性增高特征，22例（73.3％）患者AGLT50试验阳性（<290 s）；29例患者完成EOFT检测，正常对照开始溶血浓度为0.44％，完全溶血浓度为0.32％；其中23例（79.3％）开始溶血浓度升高，具体分布为0.48％者12例、0.52％者3例、0.56％者4例及0.60％者4例；完全溶血浓度升高者19例（65.5％），包括0.36％者16例及0.40％者3例。所有患者均存在明显的骨髓代偿证据：中位ARC为285.6（134.8～667.8）×10^9^/L，中位Ret％为13.5％（6.1％～29.9％），中位BMRI为194.0（121.6～307.7），中位RPI为3.5（2.2～5.1）。

三、治疗模式与疗效

图1展示了本队列的治疗模式。糖皮质激素作为所有患者的一线治疗，OR率为93.3％（28/30），含17例CR及11例PR。17例（56.7％）患者接受二线治疗，以RTX为主（9例），RTX-SD组7例中4例CR、1例PR、2例NR，RTX-LD组2例中1例CR、1例NR；余包括环孢素（4例，其中3例CR、1例PR）、雄激素（3例，其中2例CR）及硫唑嘌呤（1例CR）。6例（20.0％）患者接受后线（≥3线）治疗，其中2例糖皮质激素无效患者分别经伊布替尼及RTX-SD挽救后获得CR。伊布替尼（2例）及脾切除（1例）治疗者均获CR，另1例氟达拉滨联合RTX-SD评估为NR。后线治疗路径及转归详见表1。辅助治疗包括静脉注射丙种球蛋白（6例）、重组人EPO（3例）、来氟米特（2例）及羟氯喹（1例）。

**图1:**
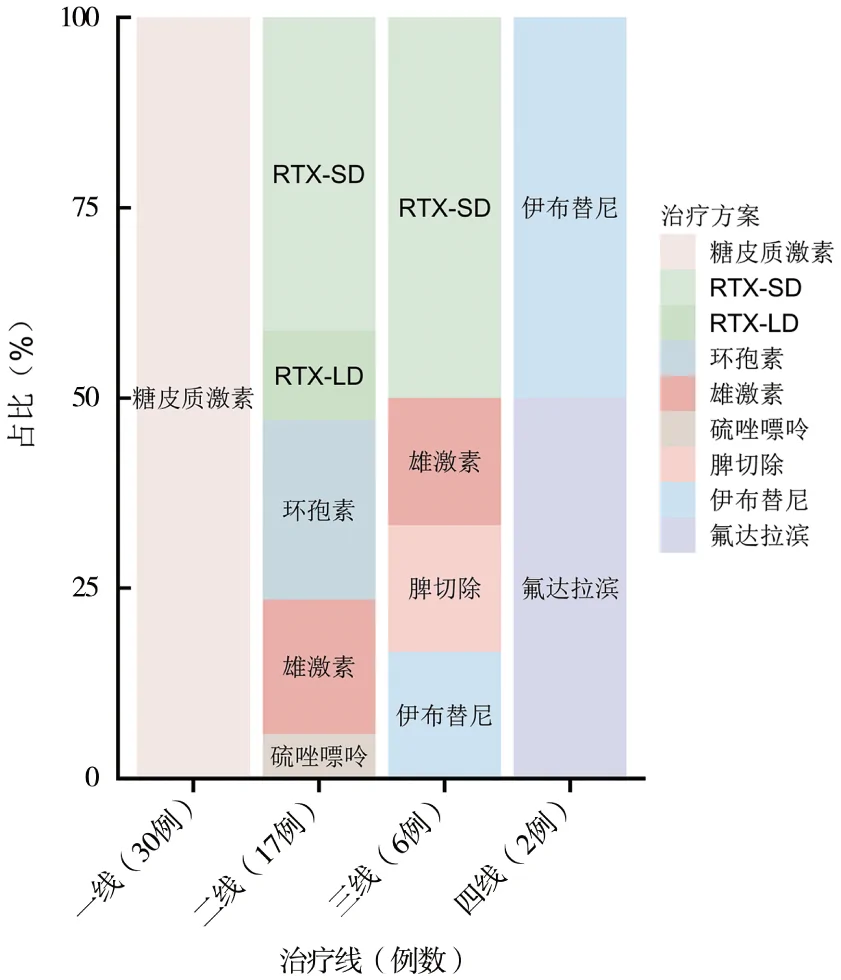
30例直接抗人球蛋白试验阴性自身免疫性溶血性贫血患者的治疗模式 **注** RTX-SD：标准剂量利妥昔单抗（每周375 mg/m^2^×4周或1 000 mg/d，第1、15天）；RTX-LD：低剂量利妥昔单抗（每周100 mg×4周）

**表1:** 6例接受三线及以上治疗的直接抗人球蛋白试验阴性自身免疫性溶血性贫血患者的治疗路径与转归

例号	一线		二线		三线		四线	
	治疗方案	疗效	治疗方案	疗效	治疗方案	疗效	治疗方案	疗效
1	GCs	NR	RTX-SD	NR	伊布替尼	CR	–	–
2	GCs	NR	环孢素	PR	RTX-SD	CR	–	–
3	GCs	PR	环孢素	CR	雄激素	CR	–	–
4	GCs	CR	RTX-LD	NR	脾切除	CR	–	–
5	GCs	PR	环孢素	CR	RTX-SD	NR	氟达拉滨	NR
6	GCs	CR	硫唑嘌呤	CR	RTX-SD	PR	伊布替尼	CR

**注** GCs：糖皮质激素；NR：未缓解；CR：完全缓解；PR：部分缓解；RTX-SD：标准剂量利妥昔单抗（每周375 mg/m^2^×4周或1 000 mg/d，第1、15天）；RTX-LD：低剂量利妥昔单抗（每周100 mg×4周）；–：不适用

四、生存与长期转归

全组中位随访时间为64（0.85～195）个月。至末次随访，全因死亡率为10.0％（3/30），死亡病例中1例死于本病进展，于确诊后第49个月死亡，另2例死因及时间不详，其末次有效随访时间分别为第10个月和第99个月。在23例可评估的存活患者中，78.3％（18/23）处于持续CR状态。余患者表现为轻度（4例）或中度（1例）贫血。由于死亡事件较少，未绘制OS生存曲线。另4例失访患者在截尾点均为存活状态。

全组中位EFS为17.0个月，1年、3年及5年EFS率分别为56.5％、45.9％和37.5％（图2）。在19例存在复发记录的患者中，73.7％的复发与药物减撤相关，其次为感染（21.1％，以呼吸道感染为主）及生理应激（10.5％，如劳累等）。此外，本组共记录2例血栓事件，均发生于溶血活动期，其中1例表现为确诊时合并的急性脑梗死；另1例于确诊10个月后并发肺栓塞，该例患者抗心磷脂抗体阳性。

**图2:**
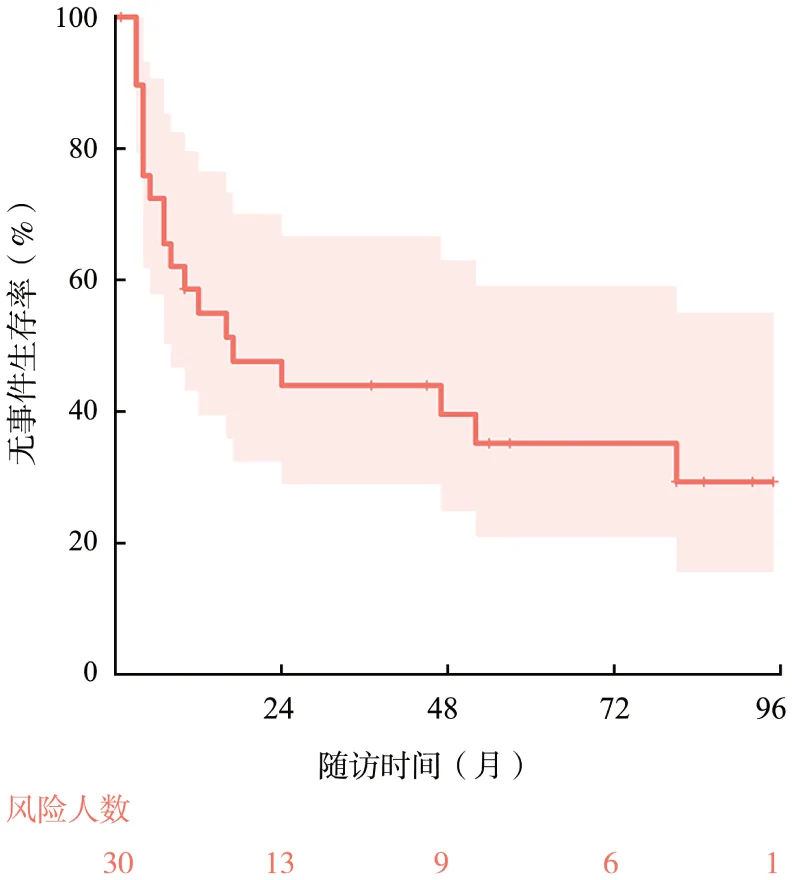
30例直接抗人球蛋白试验阴性自身免疫性溶血性贫血患者的无事件生存曲线

## 讨论

AIHA作为最常见的获得性溶血性贫血，具有显著的临床异质性。本研究纳入30例DAT阴性患者，占同期住院AIHA总数的4.77％，且女性占比更高（66.7％），与既往报道的占比基本一致[6]。但经典温抗体型AIHA（原发/继发性各50％）[1]–[2]不同，本组以原发性AIHA为主（86.7％），提示继发性AIHA可能更易呈现DAT阳性结果。既往研究亦支持不同病因温抗体型AIHA患者在自身抗体负荷方面存在差异，其中继发性较原发性患者更常出现混合型DAT（IgG+C3）模式（36.7％对17.5％）[7]。此结果与本研究中继发性DAT阴性AIHA占比较低的观察具有一致性，提示继发性AIHA可能具有更高负荷的致病抗体。然而，鉴于本研究为回顾性观察研究，相关推论仍需在大样本的前瞻性研究中进一步验证。

DAT结果的判读需结合临床审慎进行。一方面，DAT阳性并不等同于确诊AIHA。在约0.01％的健康献血者[8]及1％的住院患者（尤其是慢性淋巴细胞白血病[9]、MDS[10]、骨髓纤维化[11]）中均可见无溶血意义的阳性结果。另一方面，DAT阴性亦不能排除AIHA。尽管微柱凝胶法及流式细胞术等技术提升了检测敏感度，但受限于检测成本，并未在临床普及。本研究采取了严格的排除性诊断路径，在确诊溶血性贫血的前提下，完善红细胞酶谱及PNH克隆等检测排除经典溶血性疾病，并进一步排除了MDS、CDA及巨幼细胞性贫血等易误诊疾病[12]，确保了诊断的准确性。

关于DAT阴性AIHA的临床表型与预后转归，学术界尚存在一定分歧[2],[6],[13]。血液学检测策略的地域性差异可能会影响研究结果。欧美国家筛查常优先采用抗IgG和抗C3d单特异性抗血清，未常规覆盖抗IgA及抗IgM，易将部分非IgG介导型病例误判为DAT阴性[14]–[17]。近年来我国临床逐渐采用覆盖IgG、IgA、IgM及C3的多特异性抗血清，因此，本研究定义的DAT阴性AIHA更倾向于由低亲和力或低滴度抗体介导。部分文献将DAT阴性AIHA归入“非典型AIHA”范畴，主张一线即应启动糖皮质激素联合RTX的强化治疗策略[2],[6],[13]。日本一项大样本回顾性研究通过对比154例DAT阴性与62例DAT阳性患者，证实DAT阴性组贫血及溶血程度更为轻微，不仅糖皮质激素反应性及1年生存率与阳性组相当，且维持缓解所需的激素剂量更低[3]。本研究结果也提示由低亲和力或低滴度抗体介导的AIHA溶血过程可能更为温和。

新兴免疫抑制疗法的涌现正在逐步改变AIHA的治疗格局[13],[18]–[21]。既往常描述DAT阴性AIHA为“对糖皮质激素反应良好”[6]，现今应将其界定为“对免疫抑制治疗反应良好”的类型。本队列中2例激素治疗无效的病例，分别通过布鲁顿酪氨酸激酶抑制剂伊布替尼、环孢素序贯RTX策略使血象恢复正常。此外，针对复发诱因的分析显示，药物减撤及感染是复发的主要诱因，提示在AIHA管理中应加强患者教育，提高依从性并预防感染。

本研究局限性在于：（1）作为单中心回顾性研究且病例数仅30例，存在一定的选择偏倚；（2）由于免疫抑制治疗反应为DAT阴性AIHA的关键诊断依据之一，因此本研究对整体治疗反应率的评估可能存在一定的内在局限性；（3）整体预后转归良好，限制了预后多因素模型的构建；（4）受限于检测条件，未开展丝裂原刺激DAT等超微量抗体检测；（5）药物疗效需要审慎解读，如雄激素多为联合用药，可能反映的是与糖皮质激素的协同效应。未来仍需开展多中心前瞻性登记研究，以进一步揭示这一特殊亚型的临床全貌。

综上所述，DAT阴性AIHA是一类少见但预后相对良好的临床亚型。在完善排他性诊断及广谱Coombs分型（排除IgA/IgM介导型）的前提下，其临床经过较典型DAT阳性AIHA更为温和，对糖皮质激素及RTX等广义免疫抑制治疗反应相对理想。
